# Self-blinding citizen science to explore psychedelic microdosing

**DOI:** 10.7554/eLife.62878

**Published:** 2021-03-02

**Authors:** Balázs Szigeti, Laura Kartner, Allan Blemings, Fernando Rosas, Amanda Feilding, David J Nutt, Robin L Carhart-Harris, David Erritzoe

**Affiliations:** 1 Centre for Psychedelic Research, Imperial College LondonLondonUnited Kingdom; 2 Data Science Institute, Imperial College LondonLondonUnited Kingdom; 3 Center for Complexity Science, Imperial College LondonLondonUnited Kingdom; 4 Beckley FoundationOxfordUnited Kingdom; 5 Centre for Psychiatry, Imperial College LondonLondonUnited Kingdom; National Institute of Mental Health, National Institutes of HealthUnited States; University of MarylandUnited States

**Keywords:** psychedelics, placebo, microdosing, self-blinding, expectations, citizen science, Human

## Abstract

Microdosing is the practice of regularly using low doses of psychedelic drugs. Anecdotal reports suggest that microdosing enhances well-being and cognition; however, such accounts are potentially biased by the placebo effect. This study used a ‘self-blinding’ citizen science initiative, where participants were given online instructions on how to incorporate placebo control into their microdosing routine without clinical supervision. The study was completed by 191 participants, making it the largest placebo-controlled trial on psychedelics to-date. All psychological outcomes improved significantly from baseline to after the 4 weeks long dose period for the microdose group; however, the placebo group also improved and no significant between-groups differences were observed. Acute (emotional state, drug intensity, mood, energy, and creativity) and post-acute (anxiety) scales showed small, but significant microdose vs. placebo differences; however, these results can be explained by participants breaking blind. The findings suggest that anecdotal benefits of microdosing can be explained by the placebo effect.

## eLife digest

Psychedelic psychotherapy, therapy enhanced with psychedelic drugs such as LSD or psilocybin (the active ingredient of ‘magic mushrooms’), has been suggested to improve psychological well-being. For this reason, trials on psychedelic therapy for the treatment of depression, addiction and other conditions are ongoing. Recently, ‘microdosing’ – a way of administering psychedelics that involves taking about 10% of a recreational dose two or three times per week – has gained popularity. Unlike taking large doses of psychedelics, microdosing does not induce hallucinations, but anecdotal reports suggest that it yields similar benefits as psychedelic therapy.

A key feature of modern medicine are ‘placebo control’ studies that compare two groups of patients: one that takes a drug and another that takes inactive pills, known as placebos. Crucially, neither group knows whether they are taking drug or placebo. This control ensures that observed effects are due to the drug itself and not to unrelated psychological causes. For example, in trials of mood medicines, participants often expect to feel happier, which in itself improves their mood even when taking a placebo. This is known as the placebo effect.

Restrictive drug policies make placebo-controlled studies on psychedelics difficult and expensive, in particular for microdosing, which involves taking psychedelics over a longer time period. To overcome this problem, Szigeti et al. developed a new citizen-science approach, where microdosers implemented their own placebo control based on online instructions. The advantages are the low cost and the ability to recruit participants globally. The experiment was completed by 191 microdosers, making it the largest placebo-controlled study on psychedelics to-date, for a fraction of the cost of an equivalent clinical study.

The trial examined whether psychedelic microdosing can improve cognitive function and psychological well-being. The team found that microdosing significantly increased a number of psychological measures, such as well-being and life satisfaction. However, participants taking placebo also improved: there were no significant differences between the two groups. The findings confirmed positive anecdotes about microdosing improving people’s moods, but at the same time show that taking empty capsules, knowing they might be microdoses, have the same benefits. This result suggests that the observed benefits are not caused by the microdose, but rather by psychological expectations.

The study’s innovative ‘do-it-yourself’ approach to placebo control may serve as a template for future citizen science studies on other popular phenomena where positive expectations and social factors could play a role, such as cannabidiol (CBD) oils, nootropics and nutrition.

## Introduction

There is renewed interest in the medical application of psychedelic drugs, such as lysergic acid diethylamide (LSD) and psilocybin. Contemporary research is predominantly focusing on ‘psychedelics assisted psychotherapy’, where a few (one to three) large doses of psychedelics are used as adjunct to psychotherapy. Using this paradigm, psychedelics have shown promise in the treatment of conditions such as depression, end-of-life-anxiety, addiction, and obsessive-compulsive behaviors (Carhart-Harris and Goodwin, 2017; Nutt et al., 2020).

Recently, ‘microdosing’ has emerged as an alternative paradigm of psychedelic use. Due to its underground origin, microdosing does not have a universally agreed upon definition, and inconsistencies exist in substance, dose, frequency, and duration of use (Kuypers et al., 2019). However, microdosing can be broadly defined as the frequent use (one to three times per week) of low doses of psychedelics (10–20% of a typical ‘full’ dose, e.g. 10–15 μg LSD or 0.1–0.3 g of dried psilocybin containing mushrooms).

Anecdotal evidence suggests that microdosing may improve well-being, creativity, and cognition (Fadiman and Krob, 2017), and recent uncontrolled, observational studies have provided some empirical support for these claims (Anderson et al., 2019; Polito and Stevenson, 2019; Prochazkova et al., 2018). While encouraging, these studies are vulnerable to experimental biases, including confirmation-bias and placebo effects, in particular, because microdosers are a self-selected sample with optimistic expectations about psychedelics and microdosing (Polito and Stevenson, 2019). This positivity bias, combined with the low dose and the subjective evaluation of effects, pave the way for a strong placebo response.

A few recent double-blind, controlled studies have been conducted on microdosing. All studies used LSD and focused on the acute effects of a single microdose in a small number of healthy subjects (Yanakieva et al., 2019; Bershad et al., 2019a; Bershad et al., 2019b; Family et al., 2020; Hutten et al., 2020b). Studies have found large variability in LSD blood concentration after microdosing (Family et al., 2020), along with increased BDNF blood levels (Hutten et al., 2020a). No robust evidence was found to support the positive anecdotal claims about microdosing, but some dose-related self-rated subjective effects were detected (e.g. self-ratings of ‘*feel drug*’, ‘*feel high*’, and ‘*like drug*’) (Yanakieva et al., 2019; Bershad et al., 2019b; Hutten et al., 2020b), along with concomitant changes in brain function (Bershad et al., 2019b).

Two key issues need to be considered when assessing the scientific credibility of microdosing: the lack of placebo control in uncontrolled studies and the small sample size in controlled studies. Uncontrolled, observational studies affirm the anecdotal reports, but by design, these studies cannot provide evidence for beyond placebo benefits. Lab-based, controlled studies have small samples (Yanakieva et al., 2019; Bershad et al., 2019a; Bershad et al., 2019b; Family et al., 2020) due to restrictive drug policies that render randomized controlled trials prohibitively expensive, and hence may be statistically underpowered.

In the present study we conceived of a novel citizen-science (Silvertown, 2009) initiative as a solution to this problem, exploiting modern technology and the popularity of microdosing. The key component is a self-blinding setup procedure that enabled self-experimenters, who microdose on their own initiative using their own psychedelic, to implement placebo control and randomization without clinical supervision. To investigate potential changes over the study period, participants were directed to online self-report surveys and cognitive tasks at various timepoints. The strength of this design is that it allowed us to obtain a large sample size while implementing placebo control at minimal logistic and economic costs. The primary objective of the study was to test whether psychedelics microdosing produces superior outcomes compared to placebo on psychological state and cognitive function. We hypothesized that improvements from baseline will be positively correlated with the number of microdoses taken during the dose period and that acute/post-acute outcomes will be better under/after taking a microdose.

## Materials and methods

### Design

This study had a naturalistic design involving elements of experimental control (self-blinding), prospective data collection and online citizen-science. From baseline to the final endpoint, the study was 10 weeks long (weeks 0–9), including a core 4-week microdosing period. Primary endpoint was at week 5 and there was an optional follow-up at week 9. The self-blinding procedure randomly assigned individuals to three groups, where the groups are defined by the number of weeks taking placebos/microdoses during the dose period. The three groups were:

- Placebo (PL) group: 4 weeks of placebo,
- Half-Half (HH) group: 2 weeks of placebo and 2 weeks microdosing, and
- Microdosing (MD) group: 4 weeks of microdosing.

**Figure 1.**
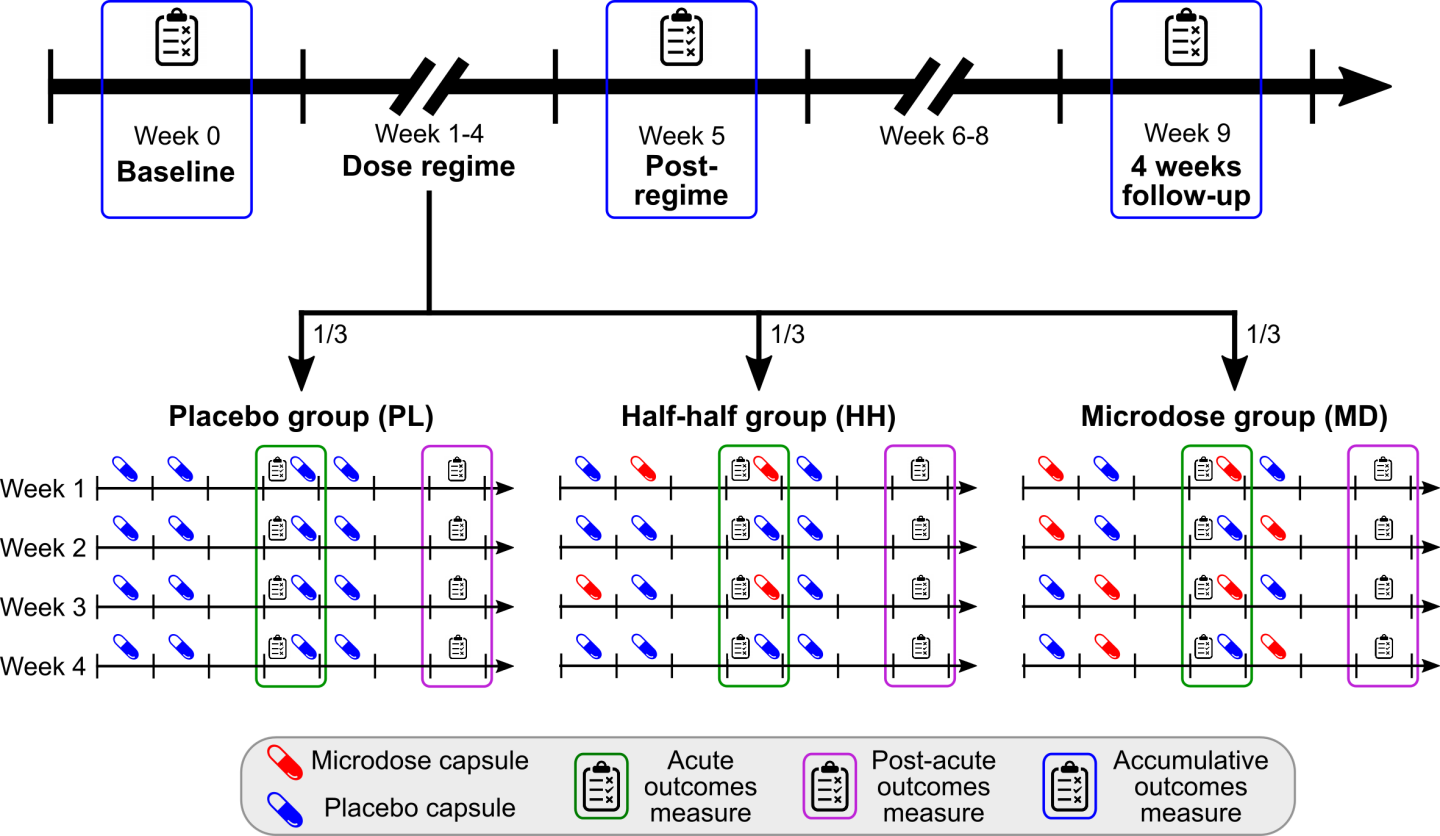
Timeline and outcomes. Top horizontal arrow shows the experimental timeline and the three timepoints associated with accumulative outcomes (blue frame). 1/3 of the participants were randomly assigned to one of the three groups, where the groups differ in the number of placebo/microdose weeks during the dose-regime: 4/0 for PL, 2/2 for HH, and 0/4 for the MD group. Note that even for microdose weeks, placebo capsules are mixed into the schedule, for example, weeks 1 and 3 for the HH group are microdose weeks. Acute measures (green frames) were taken on Thursdays, while the potential microdose was still active. Post-acute measures (purple frame) were administered on Sundays, when no capsule was taken, these outcomes test the weekly effects of microdosing. For a list of measures administered at each timepoint, see Table 1.

Individuals took two microdoses during each microdose week, resulting in 0/4/8 total microdoses for the PL/HH/MD groups. Participants had equal probability (1/3) of being assigned to each group; Figure 1 illustrates the experimental timeline and the groups’ dose schedule.

### Outcomes

Outcomes can be organized into three categories capturing the effects of microdosing on different timescales.

- Accumulative: assessed monthly, first at baseline, then after the completion of the dosing regime at week 5, and finally at the optional long-term follow-up at week 9. Accumulative outcomes were: Ryff’s psychological well-being (RPWB) (Ryff and Keyes, 1995), cognitive and affective mindfulness scale (CAMS) (Feldman et al., 2007), satisfaction with life scale (SWL) (Diener et al., 1985), green paranoid thought scales (GPTS) (Green et al., 2008), big five personality traits (B5) (McCrae and John, 1992) with the addition of *intellect* trait (DeYoung, 2015) and cognitive performance. To quantify cognitive performance, participants were tested in six tasks: spatial span, paired associates, rotations, odd one out, spatial planning, and feature match, see Hampshire et al., 2012 for details. Task scores were combined as the cognitive performance score (CPS) to quantify overall cognitive performance as a single outcome. Briefly, CPS is the average z-score across the six tasks after removing learning effects, see Appendix 1 for details.
- Post-acute: assessed weekly during the dose period on Sundays, when no capsule was taken. Measures were taken 48–72 hr after the last placebo/microdose capsule. Post-acute outcomes were: Warwick–Edinburgh mental well-being scale (WEMWB) (Tennant et al., 2007), Quick inventory of depressive symptomatology (QIDS) (Rush et al., 2003), Spielberger’s state-trait anxiety inventory (STAIT) (Spielberger, 1983), and Social connectedness scale (SCS) (Lee and Robbins, 1995).
- Acute: assessed weekly during the dose period on Thursdays, when either a microdose or placebo capsule was taken. The testing was carried out 2–6 hr after the ingestion of the capsule, while the potential microdose was active. Acute outcomes were positive and negative affect schedule (PANAS) (Watson et al., 1988), visual analogue scale items (*drug intensity, mood, energy, creativity, focus,* and *temper*) and cognitive performance (see *Accumulative* above for details).

An overview of the outcomes can be found in Table 1 and a description of each measure is in Appendix 1. See Figure 1 for the experimental timeline and assessment timepoints.

**Table 1.** List of outcomes. Outcomes have three types, depending on what is the timescale of the effect they aim to capture: *accumulative* are monthly, *post-acute* are the weekly and *acute* are the daily effects. A scale is administered at every timepoint of the associated outcome type if the checkmark is shown, for example, PANAS was administered at every acute timepoint, that is every Thursday during the dose period, see Figure 1 for a visual overview of the timepoints and see Appendix 1 for a description of each scale.

Test	Domain	Acronym	Baseline	Acute	Post-acute	Accumulative
Demographics	-	-	✔			
Previous drug experiences and expectations	-	-	✔			
Short suggestibility scale	Suggestibility	SSS	✔			
Cognitive performance score	Cognition	CPS	✔	✔		✔
Daily effects of microdosing VASs	-	-		✔		
Positive and negative affection scale	Emotional state	PANAS		✔		
Warwick–Edinburgh mental well-being scale	Well-being	WEMWB			✔	
Quick inventory of depressive symptomatology	Depression	QIDS			✔	
Social connectedness scale	Connectedness	SCS			✔	
Spielberger’s state-trait anxiety inventory	Anxiety	STAIT			✔	
Ryff’s psychological well-being scales	Well-being	RPWB	✔			✔
Cognitive and affective mindfulness scale	Mindfulness	CAMS	✔			✔
Green paranoid thought scales	Paranoia	GPTS	✔			✔
Big five personality inventory	Personality	B5	✔			✔
Satisfaction with life	Life satisfaction	SWL	✔			✔

### Self-blinding setup procedure

**Figure 2.**
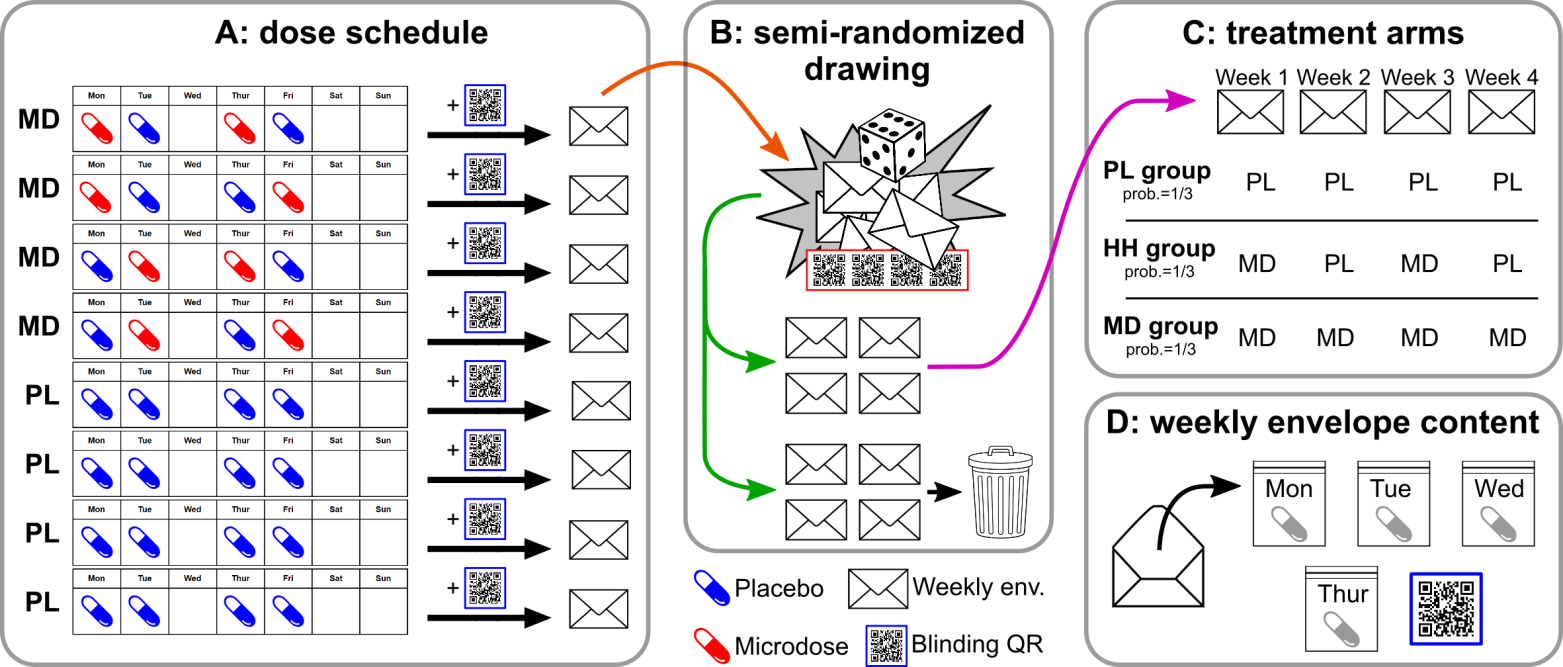
Overview of the self-blinding setup. First, capsules are prepared: microdoses are put into opaque gel capsules, while empty capsules are used as placebos. Next, weekly sets of capsules are assembled according to the dose schedule (**A**; no capsules taken on Wed., Sat., and Sun.). Then, capsules are placed inside zip bags with a printed day label (Monday, Tuesday, etc.; zip bags and day labels not shown on figure). Next, each weekly set and a unique QR code are placed inside envelopes. Eight such weekly envelopes are prepared, four of which correspond to microdose weeks (MD) and four that corresponds to placebo weeks (PL). The eight envelopes are used in a semi-random drawing process (orange arrow, **B**), which involves another set of QR codes and random number generation, see Appendix 1—figure 1 for details. The drawing selects four envelopes, corresponding to the 4 weeks of the dose period, while the remaining four are discarded (green arrow). The drawing is constrained such that only the three combinations of PL/MD weeks are possible, as shown in **C**, each with a probability of 1/3. Panel **D** shows the content of each envelope. Participants open the corresponding envelope each week and take the matching capsule every day. Scanning the QR links to the study’s IT system and enables to decode which capsule was taken when.

A high-level overview of self-blinding is provided here; for a detailed illustration see Figure 2. First, two sets of capsules had to be prepared using non-transparent capsules: one set with microdoses inside and another set without anything inside (placebos). Next, these capsules were packaged into weekly sets, which were then placed inside envelopes together with a QR code (Figure 2A). The envelopes were grouped and shuffled. Then, using a semi-random drawing process, four of them were selected (Figure 2B) corresponding to the 4 weeks of the dose period (i.e. each envelope held capsules for 1 week of the dose period). The drawing process was constrained such that only three combinations of the envelopes were possible to draw, matching the three study groups: placebo (four placebo weeks), half-half (2–2 placebo and microdose weeks), and microdose group (four microdose weeks; Figure 2C). At this stage, participants were ready to start the experiment.

When the dose period started, one envelope was opened per week and the capsules inside were used as scheduled (Figure 2D). Additionally, the QR code from the envelope had to be scanned, which shared a numeric code with our informatics infrastructure. The decryption key (i.e. how capsule types are encoded by the numbers) was not shared with participants, so the numeric code allowed only us to deduce which type of capsule was taken when.

In summary, the two key elements of self-blinding are to hide the active components inside opaque capsules while preparing identical looking placebos (1) and to position non human-readable QR codes along the capsules prior to randomization (2). With the QR codes in place, it is possible for the experimenter to recover knowledge of capsule types after randomization without revealing that information to participants.

### Microdose preparation

Participants were allowed to use any psychedelic substance to microdose with. The microdose dose, which is the amount of substance to use as a microdose, was not defined for participants, rather they were instructed to use a microdose dose that they would use outside the study. The rationale for this direction was threefold. First, given that participants typically would source their substance from the black market, the precise microdose dose could not have been known even if instructions requested it. Second, based on community feedback, most experienced microdosers have a preferred dose that they would not have liked to change to participate in the study. Lastly, this study was not a clinical trial and therefore from a regulatory perspective not allowing for control over and/or directing about drug doses.

### Recruitment and inclusion criteria

Psychedelics users were recruited through advertisement on relevant online and offline forums. Individuals could sign up through the study’s website, https://selfblinding-microdose.org/, where they could find information about the study, including the study manual and explainer videos, the participant information’s sheet, and procedure for declaring informed consent. Once informed consent was given, individuals were able to sign up by providing their email address and planned start date. The inclusion criteria were: >18 years of age, good understanding of English, intention to microdose with psychedelics, previous experience with psychedelics (either micro- or macrodosing), no use of psychedelic drugs from a week before the start until the completion of the post-regime timepoint (other than the study’s microdoses), and willingness to follow the study protocol.

### Data collection

All the questionnaires were implemented online using the SurveyGizmo platform (https://www.surveygizmo.com/). For the online assessment of cognitive performance, the Cambridge Brain Sciences (https://www.cambridgebrainsciences.com/) service was used. At each timepoint, links to each test were sent in a dedicated email via the Psychedelics Survey (https://www.psychedelicsurvey.com/) service. These links had a personal ID embedded, so each test completion could be matched to individuals.

### Blind breaking and collection of guess data

Participants were asked to guess which type of capsule they had taken that day during the dose period (for days when capsule was taken). This guess was a forced binary choice between *microdose* and *placebo* options. At the end of the post-acute test sessions, participants were asked separately to guess whether the current week was a microdose or a placebo week (Figure 1A). In the discussion of our results, the term ‘break blind’ indicates that the participant guessed the capsule correctly for the day (acute outcomes) or week (post-acute outcomes). No guess was collected about perceived group allocation at the end of study, because information about group structure was not shared with participants.

### Statistical analysis

Group differences in demographics, recreational drug use, and baseline scores of the accumulative outcomes were assessed with ANOVA and chi-square tests for continuous and categorical variables.

Accumulative outcomes were analyzed with mixed-effect repeated measurement models, using the SAS PROC MIXED method with compound symmetry covariance structure. Models were constructed with change from baseline as the dependent variable, *group*, *time* and group*time interaction as factors, and individuals as experimental unit. Models were adjusted for all significant baseline covariates (the following variables were tested as potential covariates: age, sex, education, baseline score, dose, total dose, short suggestibility scale score, expectation score, number of past psychiatric diagnosis, number of current psychiatric medications, number of lifetime macrodose experiences, and number of lifetime months microdosing). To accommodate dose as a potential covariate, psilocybin mushroom mass was converted to an estimated equivalent LSD dose (0.1 g of dried mushroom ~4.6 µg LSD; Kaplan et al., 1994; Carbonaro et al., 2016). The following planned comparisons were made: within-group comparisons of change over time from baseline to the primary endpoint at week 5 and from baseline to the final follow-up at week 9. Additionally, between-group comparisons were made (PL vs. HH and PL vs. MD) at week 5 and week 9.

To analyze acute and post-acute outcomes, mixed linear models were constructed. Models included score as dependent variable, *subject ID* as a random-effect, and *condition* as fixed-effect, where *condition* was a binary categorical variable (PL/MD). For acute outcomes, *condition* was PL/MD when the score was obtained under the influence of a placebo/microdose capsule, while for post-acute outcomes *condition* was PL/MD when the score was obtained at the end of placebo/microdose week. Planned comparisons were made between scores obtained under PL and MD conditions. Each participant contributed four scores to these models, corresponding to the four acute/post-acute assessment timepoints during the dose period. All acute/post-acute models were adjusted for all significant baseline covariates (same variables were tested for significance as in the case for the accumulative outcomes, except baseline score and total dose consumed).

To better understand how *guess* influenced scores, a second set of models were constructed with the addition of *guess* (binary categorical variable, PL/MD) and guess*condition factors. Using these guess adjusted models, planned comparisons were made between PL and MD conditions. Finally, the two binary variables (*condition* and *guess*) divided the data into 2*2 = 4 strata, post-hoc comparisons were made between the following strata (*condition/guess*): PL/PL vs. MD/PL, PL/MD vs. MD/MD, PL/PL vs. PL/MD and MD/PL vs. MD/MD. This selection was made such that *condition* changes while *guess* remains fixed in the first two comparisons, and *guess* changes while *condition* remains fixed in the last two comparisons.

### Ethical considerations

The study only engaged people who planned to microdose through their own initiative with their own psychedelic substance, but who consented to incorporate placebo control to make their self-experimentation compatible with our study. Investigators did not endorse any use of psychedelics, and no financial compensation was offered to participants. Email addresses were the only personally identifiable data collected. The email address was retained after study completion if permission was given (checkbox) by the participant to receive information regarding future studies, discarded otherwise. The study was approved by Imperial College Research Ethics Committee and the Joint Research Compliance Office at Imperial College London (ICREC reference number 18IC4518).

## Results

### Demographics, randomization, and completion rate

**Figure 3.**
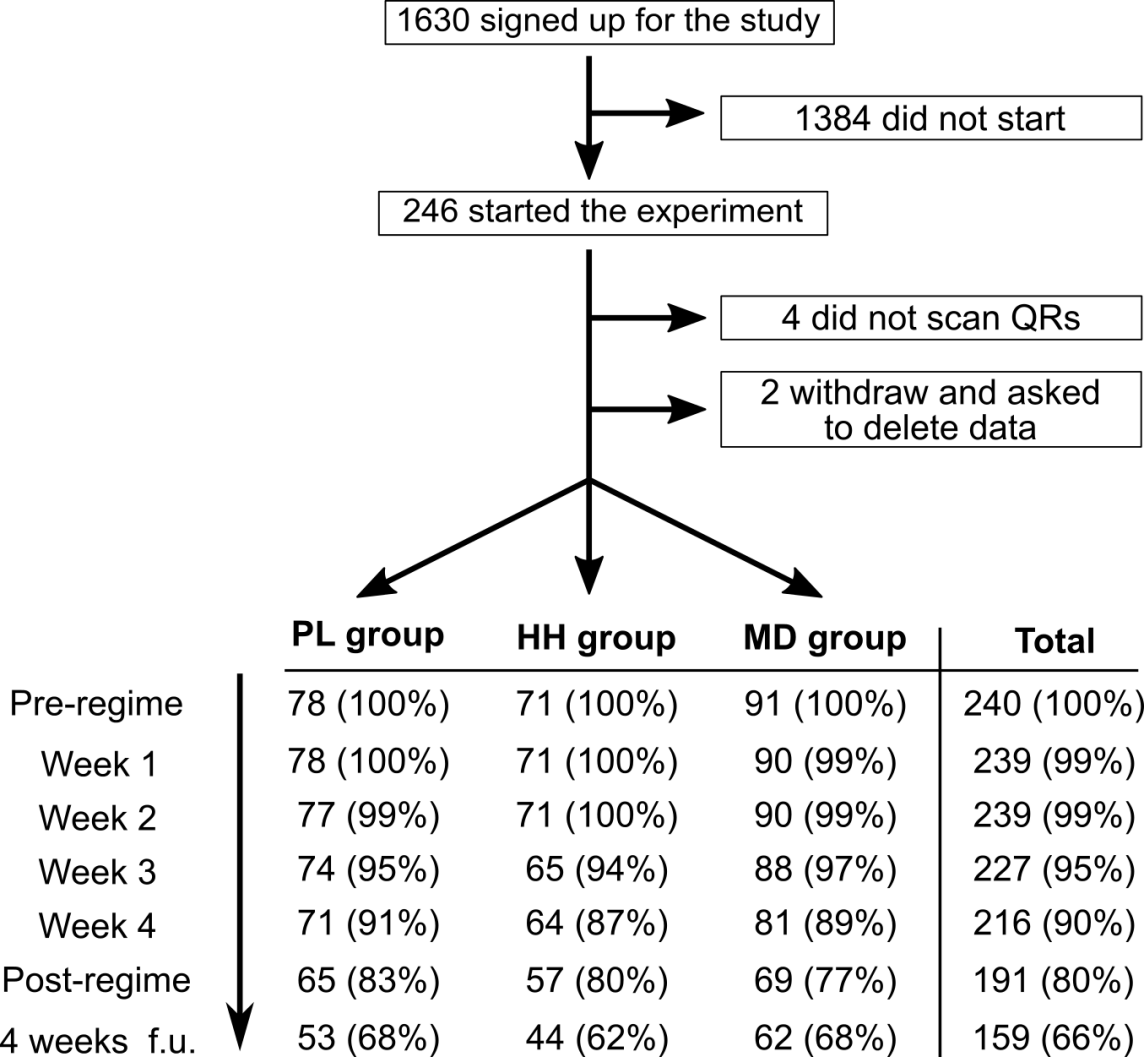
Flow diagram showing participation and completion rates through the study. The completion of the 4 weeks follow-up timepoint was optional.

A total of 1630 participants signed-up, 240 started, and 191 participants completed the study. The optional follow-up at week 9 was completed by 159 individuals. No statistically significant differences were found between the groups in any demographic, recreational drug use or baseline measures, confirming efficiency of the randomization (see Supplementary file 1 for details on demographics, Supplementary file 2 for recreational drug use, and Supplementary file 3 for statistical analysis of baseline variables). Completion rate was highly similar across the three groups (*χ^2^*(12, *N* = 240)=0.64, p=0.99), see Figure 3.

For the most part, the sample consisted of educated, middle-age (33.5 ± 9.4), healthy males (70% male, 19% female, 1% other) from western countries. As expected, most participants had a positive attitude toward psychedelic drugs, in particular toward medical use: 74% and 90% either *agreed* or *strongly agreed* with the statements *'I am an active advocate of psychedelic drug-use'* and *'I am an active advocate of the therapeutic use of psychedelics'*, respectively. See Appendix for details on the sample’s expectations/attitude about microdosing and psychedelics. The sample consisted of healthy individuals for the most part: 33% of participants reported to have had at least one psychiatric diagnosis in the past, the most frequent past diagnoses were: anxiety disorder (13%), depression (13%), and PTSD (7%). Only 7% of the sample had current mental diagnosis.

### Microdoses

Most participants microdosed with LSD (n = 147; 61%)/LSD analogue (n = 33; 14%), followed by psilocybin containing mushrooms (n = 57; 24%) and three individuals used other psychedelics (LSA: n = 1; DOB: n = 2). The average reported dose for LSD/LSD analogues was 13 ± 5.5 µg, while for psilocybin mushroom it was 0.2 ± 0.12 g, see Appendix 1—figure 3 for further details.

### Accumulative outcomes

Accumulative outcomes were first collected at baseline, then at week 5 (i.e. after the completion of the 4 weeks long dose period) and at the optional long-term follow-up timepoint at week 9. The following two sets of pre-planned comparisons were made: within group comparisons of baseline vs. week 5, baseline vs. week 9 (changes over time) and between-group comparisons at the week 5 and week 9 timepoints. Sample sizes were n = 240/191/159 at baseline, week 5 and week 9, respectively. Data was also analyzed separately for LSD/LSD-analogues and psilocybin microdoses, the results from both subgroups matched the results of the combined analysis presented here.

For the within group (change over time) comparison of baseline vs. week 5, all self-reported psychological outcomes improved significantly in the MD group: *well-being* (RPWB) increased with 4.2 ± 3.9 (adjusted mean estimate ±95% CI; p=0.04*), *mindfulness* (CAMS) increased with 2.4 ± 1.1 (p<0.001***), *life satisfaction* (SWL) increased with 1.2 ± 1.2 (p=0.04*), and *paranoia* (GPTS) decreased with −5.0 ± 1.7 (p<0.001***). Personality structure (B5) showed reduced *neuroticism* trait score (−1.3 ± 0.9, p<0.01**) and increased *openness* (0.9 ± 0.8, p=0.03*). Significant changes over the same period (from baseline to week 5) were also observed in the PL and HH groups for *mindfulness* (PL: 1.6 ± 1.1, p<0.01**; HH: 1.3 ± 1.2, p=0.02*) and *paranoia* (PL: −3.4 ± 1.7 p<0.001***; HH: −4.9 ± 1.9 p<0.001***), but not for *well-being* or *life satisfaction. Neuroticism* also decreased in the PL group (−1.0 ± 1.0, p=0.04*). Changes in *mindfulness* and *paranoia* were sustained at the week 9 follow-up timepoint for all groups, while decreased *neuroticism* only prolonged in the MD group, see Supplementary file 5 for details. CPS did not change in the MD group (from baseline to week 5), but significantly decreased in the HH group (−0.16 ± 0.14, p=0.03*). Among individual cognitive tests over the same period, *rotations* (0.34 ± 0.28, p=0.02*) and *odd one out* (0.52 ± 0.31, p=0.001**) increased significantly in the MD group, while *spatial span* (−0.49 ± 0.30, p=0.02*) and *paired associates* (−0.51 ± 0.30, p=0.02*) decreased in the HH group. The increased *rotations* score in the MD group was sustained at the follow-up (0.45 ± 0.46, p<0.01**), but not the other task scores.

**Figure 4.**
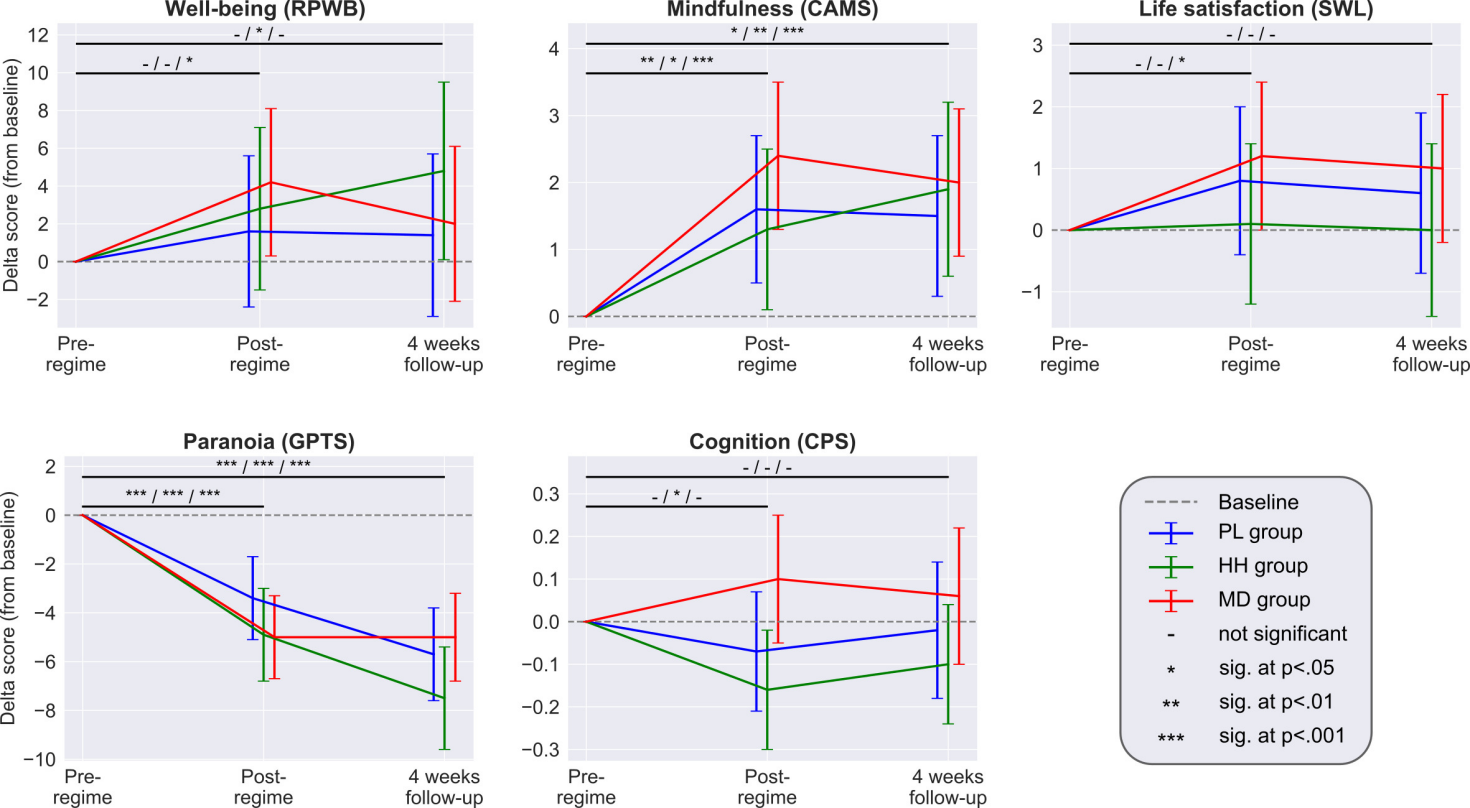
Each panel shows the adjusted mean estimate of the change from baseline and the 95% CI for the accumulative outcomes. Top horizontal bars represent the over time comparisons for each group (from baseline to post-regime [week 5] and from baseline to follow-up). Symbols on top of bars show the significance for the PL/HH/MD groups, respectively (e.g. change from baseline to post-regime in well-being was significant for the MD group, but not significant for the other two groups, see legend). There was no significant between-groups difference at any timepoint for any scale. Sample size was 240/191/159 at the pre-, post-regime and 4 weeks follow-up timepoints, respectively. See Supplementary files 4, 5, and 6 for the unadjusted descriptive statistics, adjusted mean differences (and their significance) associated with both over time and between group comparisons and model parameters, respectively.

Planned comparisons revealed no significant between-group differences at either the week 5 or week 9 follow-up timepoints, including all subscales, except that in the HH group the *paired associates* scores decreased (PL vs HH adjusted treatment difference: −0.55 ± 0.43, p<0.01**). Time course of the adjusted mean estimates is summarized in Figure 4. See Supplementary file 4 for descriptive statistics, including subscale and individual cognitive test scores, adjusted over time and between group differences (Supplementary file 5), and model parameters (Supplementary file 6).

### Accumulative outcomes adjusted for number of microdose guesses

As secondary analysis to further examine the role of placebo-like expectation effects in the accumulative outcomes, we performed a post-hoc adjustment by adding the *‘number of times microdose capsule was guessed’* variable as a covariate to the models (irrespective whether the guess was correct or not). This variable was significant for some models (RPWB: p<0.01**; CAMS: p=0.02*; B5 agreeableness: p=0.02*; B5 openness: p=0.03*) and further decreased the already small between-group differences on self-reported scales, while it did not affect cognitive outcomes. Specifically, the adjusted treatment difference (±95% CI) at the week 5 timepoint between PL and MD groups without/with the number of MD guesses covariate was: well-being (RPWB) 2.5 ± 5.6 (p=0.37)/0.9 ± 5.7 (p=0.76), mindfulness (CAMS): 0.8 ± 1.5 (p=0.32)/0.4 ± 1.5 (p=0.65), paranoia (GPTS): −1.6 ± 2.5 (p=0.21)/−1.2 ± 2.5 (p=0.36), life satisfaction (SWL) 0.4 ± 1.7 (p=0.67)/0.2 ± 1.8 (p=0.83), B5 intellect: −0.2 ± 1.2 (p=0.80)/−0.2 ± 1.2 (p=0.71), B5 openness: 0.3 ± 1.2 (p=0.57)/0.0 ± 1.2 (p=0.97), B5 neuroticism: −0.3 ± 1.4 (p=0.70)/−0.1 ± 1.4 (p=0.87), B5 extraversion: −0.2 ± 1.2 (p=0.81)/−0.4 ± 1.3 (p=0.52), B5 agreeableness: 0.5 ± 1.1 (p=0.37)/0.2 ± 1.1 (p=0.75), and B5 consciousness: 0.8 ± 1.3 (p=0.24)/0.5 ± 1.3 (p=0.44).

### Acute and post-acute outcomes

First, outcomes are described without considering the guess component, which is discussed in the next section. Acute outcomes were measured during the dose period while the potential microdose was still active, while post-acute outcomes were measured every Sunday, when no capsule was taken, 48–72 hr after the last placebo/microdose capsule. For psychological measures the average sample size was 857 (between 849 and 884 due to partial completions; participants contributed four scores corresponding to the four acute timepoints, see Materials and methods for details), while for cognitive performance it was 684 (between 678 and 689). Data was also analyzed separately for LSD/LSD-analogues and psilocybin microdoses, and the results from both subgroups matched the results of the combined analysis presented here.

Among acute measures, *condition* (PL vs. MD) was significant for acute *emotional state* (PANAS) (adjusted mean estimate ±95% CI: 2.2 ± 1.4, p<0.01**) and the acute *drug intensity* (12.5 ± 3.0, p<0.001***), *mood* (4.6 ± 2.9, p<0.001***), *energy* (5.3 ± 2.7, p<0.001***), and *creativity* (4.7 ± 2.6, p<0.001***) VASs, meaning that scores collected on days when a microdose was taken were significantly higher compared to scores collected on placebo days. Effect sizes, as quantified by Cohen’s *d*, remained small (*d* < 0.3) on all scales, with the exception of the *drug intensity* VAS (*d* = 0.58).

Among post-acute measures, *condition* was significant only on the anxiety measure (STAIT; −1.4 ± 1.3, p=0.03*), meaning that anxiety was reduced at the end of microdose weeks compared with placebo weeks, see Table 2 for details on both acute and post-acute outcomes.

**Table 2.** Summary of acute and post-acute outcomes. Acute outcomes were measured on dosing days (Thursdays), while the potential microdose was still active, comparison is made between scores obtained under the influence of microdose vs placebo capsules. Post-acute outcomes were measured at the end of the weeks (Sundays), when no capsule was taken, and comparison is made between scores obtained at the end of placebo weeks vs microdose weeks. For the psychological measures (all except CPS) the sample size was 857 (participants contributed four scores corresponding to the four acute/post-acute assessment timepoints during the dose period), while for cognitive performance it was 684. The first three columns show the unadjusted, observed scores and Cohen’s *d* between the two conditions (PL/MD). In the next column, results from the models without the guess component are shown, and last column shows model results with the guess component, each cell shows the adjusted mean difference ±95% CI of *condition* (PL vs. MD, where PL is used as baseline), see Materials and methods for details. Individual subscales/sub-tasks are shown when they exist (in the Test column, ‘*X – y*’ denotes that *y* is a subscale or sub-test of *X*).

	Observed scores			Model wo. guess	Model with guess
Test	PL (M ± 95% CI)	MD (M ± 95% CI)	Cohen's *d*	M ± 95% CI	M ± 95% CI
Acute outcomes					
Acute mood (PANAS)	14.2 ± 0.9	16.2 ± 1.1	0.19	2.2 ± 1.4**	0.9 ± 1.4
PANAS – positive	29.9 ± 0.7	31.6 ± 0.8	0.22	2.0 ± 1.0***	0.8 ± 1.0
PANAS – negative	15.7 ± 0.5	15.4 ± 0.5	−0.06	−0.4 ± 0.7	−0.3 ± 0.8
Daily effects VAS – intensity	8.5 ± 1.6	21.4 ± 2.7	0.58	12.5 ± 3.0***	3.4 ± 2.0***
Daily effects VAS – energy	55.3 ± 1.8	60.7 ± 2.1	0.27	5.3 ± 2.7***	2.4 ± 2.8
Daily effects VAS – mood	60.5 ± 1.8	64.7 ± 2.2	0.20	4.6 ± 2.9***	1.5 ± 2.8
Daily effects VAS – creativity	53.5 ± 1.6	58.3 ± 2.0	0.25	4.7 ± 2.6***	1.8 ± 2.6
Daily effects VAS – focus	57.3 ± 1.7	58.7 ± 2.1	0.07	1.3 ± 2.8	−0.6 ± 2.8
Daily effects VAS – temper	36.5 ± 2.0	36.0 ± 2.5	−0.02	−1.3 ± 3.2	0.1 ± 3.2
Cognition (CPS)	−0.08 ± 0.05	−0.06 ± 0.07	0.05	0.05 ± 0.08	0.04 ± 0.07
CPS – rotations	−0.09 ± 0.09	−0.06 ± 0.11	0.03	0.11 ± 0.14	0.12 ± 0.14
CPS – odd one out	0.02 ± 0.11	0.14 ± 0.1	0.12	0.09 ± 0.16	0.08 ± 0.16
CPS – spatial planning	−0.11 ± 0.1	−0.1 ± 0.11	0.00	0.04 ± 0.14	0.03 ± 0.14
CPS – spatial span	−0.18 ± 0.09	−0.18 ± 0.11	0.00	0.01 ± 0.14	0.02 ± 0.14
CPS – feature match	−0.06 ± 0.09	−0.05 ± 0.13	0.00	−0.05 ± 0.15	−0.07 ± 0.16
CPS – paired associates	−0.1 ± 0.09	−0.11 ± 0.12	−0.02	0.05 ± 0.15	0.05 ± 0.15
Post-acute outcomes					
Mental well-being (WEMWB)	49.7 ± 0.8	49.8 ± 0.7	0.02	0.9 ± 1.0	0.1 ± 1.0
Depression (QIDS)	5.6 ± 0.5	5.5 ± 0.4	−0.03	−0.3 ± 0.6	−0.1 ± 0.6
Anxiety trait (STAI-T)	38.5 ± 1.2	38.1 ± 1.0	−0.04	−1.5 ± 1.3*	−0.1 ± 0.6
Social conn. (SCS)	32.1 ± 0.7	32.1 ± 0.6	0.00	0.1 ± 0.8	−0.3 ± 0.8

*=p<0.05; **=p<0.01; ***=p<0.001.

### Association between guess and acute/post-acute outcomes

Next, the acute and post-acute results were re-analyzed with the addition of *guess* into the models. *Condition* (PL vs. MD) was no longer significant for any scale, except for acute *drug intensity* VAS (adjusted mean difference ±95% CI: 3.4 ± 2.0; p<0.001***), which increased under MD (Table 2). The guess*condition interaction term was non-significant for all scales, except for *drug intensity* (p<0.01**).

**Figure 5.**
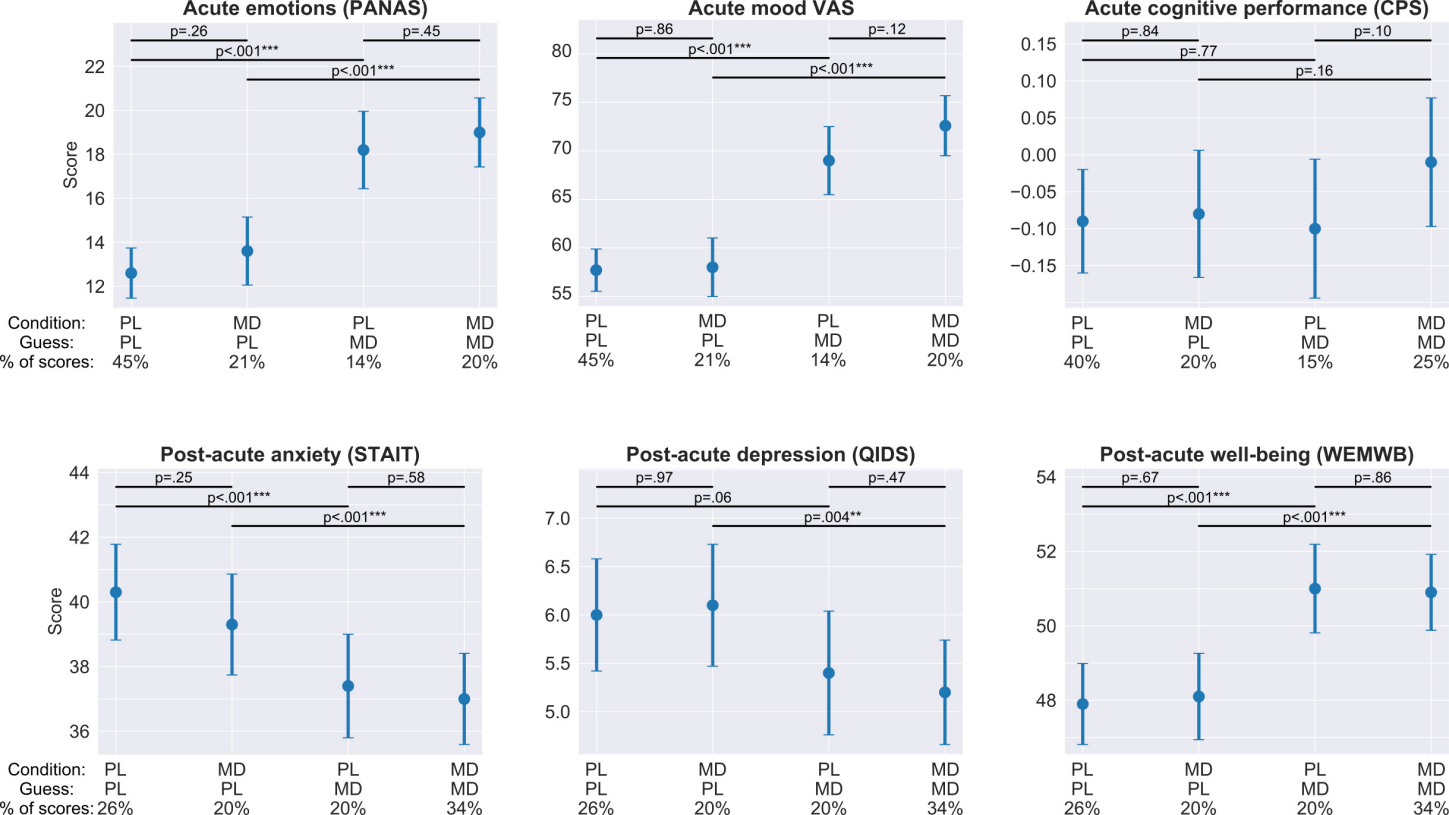
Acute and post-acute outcomes stratified by guess and condition. On each panel, the four bars represent the adjusted mean estimates and the associated 95% CI of the four strata corresponding to the four combinations of guess (PL/MD) and condition (PL/MD). For the psychological measures (all, but CPS) the sample size was 857 (participants contributed four scores corresponding to the four acute/post-acute assessment timepoints during the dose period), while for cognitive performance it was 684, see bottom of each panel for the condition, guess, and the proportion of scores in the given strata. Top horizontal lines represent comparisons between strata derived from the models. The two short lines on top are the comparisons between PL and MD conditions with fixed guess, while the two longer lines below are the comparisons between PL and MD guesses with fixed drug condition, see Supplementary file 8 for numerical results. Note that for all self-reported outcomes, change in guess is almost always significant, while a change in condition is never significant. In the case of cognitive performance, neither change in guess nor change in condition is significant.

To better understand the role of *guess*, the data was further analyzed by comparing the 2*2 = 4 strata formed by the two binary variables, *condition* (PL/MD), and *guess* (PL/MD), in the models. For self-reported outcomes, no significant differences were found between microdose and placebo conditions with fixed guess (*condition*/*guess*: PL/PL vs. MD/PL and PL/MD vs. MD/MD comparisons), except for acute *drug intensity* visual analogue scale, which was higher when microdose was taken (adj. mean difference ±95% CI; 7.3 ± 3.1, p<0.001***). Conversely, when drug condition was fixed (*condition*/*guess*: PL/PL vs. PL/MD and MD/PL vs. MD/MD comparisons), significant differences were found in 21 of the 22 comparisons (=2*conditions*(4*post-acute+7*acute scales)), all favoring MD guess. These findings suggest that scores are significantly better when the participant believed they had taken a microdose irrespective of what was actually taken. Taking an actual microdose was only associated with a significant difference in the *drug intensity* scale. Figure 5 shows the stratified distribution of selected outcomes, see Supplementary file 8 for all comparisons.

### Blinding integrity

Break blind rate, defined as the proportion of correct capsule guesses (see section *Blind breaking and collection of guess data* for details), was 0.72 ± 0.18 (M ± SD). S*pecificity* (true negative rate: ratio of true placebo guesses to all placebo guesses) was 0.82 ± 0.16, noticeably higher than *sensitivity* (true positive rate: ratio of true microdose guesses to all microdose guesses) 0.45 ± 0.30, meaning that placebo capsules were guessed correctly at a higher rate than microdoses. Based on knowledge of the ratio of PL/MD capsules (3/1) in the envelopes, which is evident to participants when they prepare the capsules, a ‘random guesser’ would have a break blind rate of 0.62 with 0.75 *specificity* and 0.25 *sensitivity*. The high sensitivity exhibited by participants (0.46 vs. the random guesser’s 0.25) suggests that the higher than random break blind rate is mostly due to superior ability to identify microdoses, see Appendix 1—table 1 for details.

Break blind rate was positively associated with reported microdose dose (*F*(1, 237)=7.4, p<0.01**), meaning that the higher the dose was, the more likely participants guessed their daily condition correctly. For this analysis psilocybin mushroom doses were converted to estimated LSD dose equivalent, see Statistical analysis in Materials and methods for details. The estimated ‘detection threshold’, that is, the dose above which participants guess significantly better than random, was 12 µg.

## Discussion

We employed a novel self-blinding methodology to investigate the acute, post-acute, and long-term, accumulative effects of psychedelic microdosing. To the best of our knowledge, this study is the first one to use a self-blinding methodology, the first placebo-controlled investigation of the accumulative effects of repeated microdosing, and the largest placebo-controlled psychedelic study to-date.

When looking at changes over time from baseline to week 5 (accumulative outcomes) in the microdose group alone, results confirmed the psychological benefits reported by anecdotes (Fadiman and Krob, 2017) and observational, uncontrolled studies (Anderson et al., 2019; Polito and Stevenson, 2019; Prochazkova et al., 2018): significant improvements were observed in the domains of well-being, mindfulness, life satisfaction, and paranoia. However, when looking at the between-group comparisons of the same outcomes, no significant differences were found between the placebo and microdose groups. On the cognitive tests, which are less subjective than the self-reported psychological outcomes, the microdose group did not even improve from baseline to week 5 and the between-groups comparisons were not significant either. Thus, our study validates the positive anecdotal reports about the psychological benefits of microdosing (significant improvements from baseline in a broad range of psychological measures); however, our results also suggest that these improvements are not due to the pharmacological action of microdosing, but are rather explained by the placebo effect (lack of significant between-groups differences).

Similar conclusions can be drawn from the examination of the acute and post-acute outcomes as well. In our initial analysis without incorporation of the guess component, we detected significant effects on post-acute *anxiety* (STAIT), acute *emotional state* (PANAS), and *mood*, *energy*, *creativity*, and *drug intensity* (visual analogue scale items). Effect sizes were small on all scales (Cohen’s d < 0.3 except *drug intensity*); thus, the clinical and practical value of these effects is debatable. Furthermore, when the guess component was added to the models, the already small differences disappeared on all scales, except for acute *drug intensity*. It can be argued that the addition of the guess variable to the models may undermine the statistical significance of the condition effect due to collinearity between condition and guess. To overcome this potential issue, we conducted the stratification analysis where only one of these variables is changing, while the other remains fixed. No significant differences were observed between placebo and microdose conditions when the guess was fixed (*condition/guess*; PL/PL vs. MD/PL and PL/MD vs. MD/MD comparisons), except for *drug intensity* (MD>PL). Conversely, when condition was fixed (PL/PL vs. PL/MD and MD/PL vs. MD/MD comparisons), scores obtained under placebo and microdose guesses were significantly different in 21 out of the 22 comparisons, always favoring the microdose guess, see Figure 5 and Supplementary file 8. Importantly, neither CPS nor any cognitive subtask, the non-self-rated outcomes where beliefs and subjective feelings are likely to be less influential, were significantly different under either guess or drug conditions. In summary, these results strongly suggest that the actual content of capsules did not determine differences between the conditions, but *beliefs* about their content did.

An important observation was that participants guessed their capsules correctly in 72% of the cases. This break blind rate was higher than random (random: 63% vs. participants: 72%), but not as high as reported in antidepressant studies (around 80%) (Chen et al., 2011; Kirsch, 2019; Rabkin et al., 1986). It is known from a variety of clinical studies that higher break blind rate is associated with larger between-conditions effect-sizes (where placebo is the control condition) (Baethge et al., 2013; Berna et al., 2017; Laferton et al., 2018). This relationship is explained by non-specific treatment factors such as expectation of a benefit (Bausell, 2009) and investigator alliance (Chatoor and Kurpnick, 2001). The influence of such factors is likely to be large for the present study, because of highly positive expectations and favorable attitudes toward psychedelics, see attitude analysis in the Appendix. These factors together suggest that the observed ‘significant’ acute and post-acute effects may be an artifact of the combination of break blinds and expected benefits. The acute and post-acute results observed could be understood as the difference between the expected benefits when a microdose is perceived (i.e. guessed by participants) versus the absence of expected benefits when placebo is perceived. This difference in expectations could be mistaken for a ‘real’ drug effect in any study where blinding integrity is not considered during analysis. If this explanation is correct, one prediction for future microdose studies with a similarly pro-psychedelics sample is that they may observe larger effects when break blind rate is higher, or conversely, smaller effects when break blind rate is lower.

What factors account for the blind breaking? *Drug intensity* was the only outcome that remained significant even after adjusting for guess (3.4 ± 2.0; p<0.001***). This observation suggests that *drug intensity* is a small, but true drug effect. This increased drug intensity mostly manifested as body and perceptual sensations, see *Blind breaking cues* in Appendix 1 for details. This finding suggests that in most cases blind breaking induced clinically irrelevant side effects, rather than deduced from improvements of outcome variables. We note that according to our data the threshold LSD dose where participants guess better than random is 12 µg, see Figure 4, which is in line with the 13 µg threshold dose estimated by a recent dose controlled study (Bershad et al., 2019a).

It is worth noting that the current study was designed to protect blinding integrity by including placebos for the microdose group as well, administering the microdose capsules on different days of the week and by including the half-half group. The 3-arm design can be seen as a strength in this regard, adding ambiguity and thus strengthening blinding. Illustrative of the integrity of the blind, we received several emails from participants in the PL group who were in disbelief after opening their unused envelopes containing unused capsules after the conclusion of the study:

- “I counted the number of cut blotters I had in the left overs: they are 8...so you must be right... Which is incredible […] Some days during the test were really, really focused and colours more vivid. This sensation was really new to me".
- "I have just checked the remaining envelopes and it appears that I was indeed taking placebos throughout the trial. I'm quite astonished […] It seems I was able to generate a powerful 'altered consciousness' experience based only the expectation around the possibility of a microdose".
- "An empty pill with strong belief/intentions makes nearly everything. You put spirituality into an empty pill here...wow!"

### Limitations

It is our view that the present part-controlled, part-observational design yields data superior to conventional observational data (inclusion of placebo control), but inferior to controlled clinical trial data (incomplete control over recruitment, screening, assessment, drug administration, etc.). This study does, however, have greater ecological validity than would a fully controlled lab study.

A key limitation of the present study is the lack of verification of the nature, purity, and dosage of the psychedelic substance used for microdosing. Psilocybin-containing mushrooms were used by 23% of the sample, 14% used legal LSD analogues (such as 1P-LSD), whereas 62% sourced their substance from the black market, mostly LSD (61%). According to the Energy Control's drug checking service (Barcelona), LSD blotter adulteration rates were low during the period when our study was running: in both 2018 and 2019 blotters sold as LSD contained LSD only in 90% (n = 735) of tested samples [personal communication with M. Ventrua from EC, June 2020]. The exact quantity of active ingredient within a given microdose cannot be known with certainty; however, the positive relationship between dose and blind breaking (Figure 4) and that the threshold dose for psychoactivity was consistent with a recent controlled study (12 µg vs 13 µg; Bershad et al., 2019a) provide some reassurance. Nonetheless, our results should be not understood as clinical evidence, rather they are representative of ‘real life microdosing’.

We could not confirm whether participants followed accurately the self-blinding procedure. Three individuals reported following an invalid sequence of weeks, but these individuals did their setups together, all committing the same mistake (1.3% error rate). Furthermore, we had no way of confirming whether the capsules were taken as instructed during the dose period. Instructions emphasized not to complete assessments planned on dosing days in case the dose schedule could not be followed for any reason, but we could not confirm whether participants adhered to this rule.

Our stratification analysis does not allow for a strict determination of a causal relationship between guess and outcome, because guess was recorded *after* completion of assessments, guess was last question during test sessions. After closing the study, a survey was conducted among participants, where 86% (n = 166) responded that "I was thinking about whether I took a microdose or placebo even before I was asked to guess" (opposed to "I was not thinking about whether I took a microdose or placebo, except when I was asked to guess"), making a causal interpretation more likely. We note that the order we chose is consistent with previous work in psychiatric studies (Baethge et al., 2013; Chen et al., 2011; Rabkin et al., 1986); had the guesses been requested prior to the assessments, it could have primed responses. Also, we cannot rule out that performance during the assessments influenced the guess. However, the lack of any feedback from the assessments mitigates this risk. Most participants reported to break blind due to body and perceptual sensations, rather than improved outcomes, see *Blind breaking cues* in the Appendix for details.

We cannot rule out the possibility that a study in a clinical population would yield more promising results. In the present healthy sample, where well-being scores are high at baseline, there is less scope for potential improvements, which could have prevented the observation of placebo-microdose differences. Most study participants reported not to have any history of mental health problems; only 7% reported having a current psychiatric diagnosis, and 33% reported to have had a psychiatric diagnosis in the past (Supplementary file 1). We conducted two post-hoc analysis for two selective pseudo-depression subsamples: participants with the lowest 25% baseline well-being scores and those with the highest 25% baseline neuroticism scores (Ryff and Keyes, 1995; Wood and Joseph, 2010). Results in these subsamples were entirely consistent with those from the complete sample: there were no significant differences between conditions for any of the accumulative outcomes (adjusted treatment difference ±95% CI of PL vs MD at week 5 for the lowest 25% baseline well-being subsample: well-being (RPWB) −1.6 ± 13.6 (p=0.81), mindfulness (CAMS) 0.3 ± 3.3 (p=0.85), paranoia (GPTS) −5.1 ± 6.8 (p=0.14), life satisfaction (SWL) 0.3 ± 4.5 (p=0.87), cognition (CPS) 0.1 ± 0.55 (p=0.71); same measures for the highest 25% baseline neuroticism subsample: well-being (RPWB) 4.8 ± 14.3 (p=0.50), mindfulness (CAMS) 1.3 ± 3.7 (p=0.49), paranoia (GPTS) −3.1 ± 8 (p=0.43), life satisfaction (SWL) −1.4 ± 4.6 (p=0.53), cognition (CPS) 0.04 ± 0.67 (p=0.90)). Thus, although not designed as a clinical study, data from this opportunistic naturalistic study do not provide support for clinical effects of microdosing.

Although this was the largest placebo-controlled psychedelic research study published to-date, we note that one could argue that the study was still underpowered to detect a true effect based on the fact that the MD group did improve more than the PL group on all scales (from baseline to week 5), but just not to a statistically significant extent (Figure 4). On the well-being scale (RPWB), the adjusted PL vs. MD group difference was 2.5 ± 5.6 points. To illustrate this difference in practice, this scale consists of 42 statements that participants rate on a 6-point Likert scale (*Strongly disagree - Strongly agree*), thus, the full range of scores is thus 0–252, so the 2.5 point mean difference is 1% of the total scale. This difference is equivalent to scoring one item, for *example ‘I like most aspects of my personality’*, *Strongly agree* instead of *Slightly agree* or *Slightly disagree*, while responding the same to the remaining 41 items. Based on our data, we calculated that the sample size (90% power and alpha of 0.05) required to observe a true between-group difference would be: 1508 for *well-being* (RPWB), 1638 for *mindfulness* (CAMS), 4918 for *life satisfaction* (SWL), 1392 for *paranoia* (GPTS), and 366 for *cognitive performance* (CPS). These differences therefore are not clinically meaningful or sufficient to justify the cost of intervention.

### Future directions

The successful execution of this initiative here may inspire similar initiatives throughout the world in a broad range of scientific and medical contexts. Controlling for placebo effects is important for trending phenomena, such as cannabidiol (CBD) oils, nootropics, and nutrition, where social-pressure, expectancy, positive-test strategies, and confirmation bias can lead to false-positive findings. Self-blinding citizen-science initiatives could be employed in these areas as a cost-efficient screening tool prior to conducting expensive clinical studies.

An important feature of the self-blinding methodology is the low cost; we estimate that the current study’s costs were about 0.5–1% of an equivalent clinical study. Since the research team is not providing the study drug/placebo and on-site staffing is not required, expenses are similar to a conventional observational study, yet still with incorporation of randomization and placebo control.

Important lessons can be taken from the current study for the design of future microdosing trials. The combination of the lack of detected efficacy in this study and an association between self-reported doses and ability to break blind (see Figure 4) suggest that selecting dosage is fraught with difficulties: if a low microdose is chosen, efficacy is unlikely if we extrapolate current results, whereas a high microdose could jeopardize the blinding. Randomization to microdose versus an active placebo conditions (e.g. niacin, which has been employed in macrodosing studies Ross et al., 2016) and careful assessment of blinding could, in principle, alleviate some of these concerns.

The present study also has implications for full/‘macrodose’ psychedelic studies, where blinding is impossible due to the intense nature of the experience. It can be hypothesized that the intense hallucinations are essential for therapeutic outcome (Griffiths et al., 2011; Roseman et al., 2017), questioning the suitability of placebo-controlled trials in this context. The fact that one may be unable to fully extricate belief, or ‘context’ more broadly, from the direct (e.g. pharmacological) action of a given intervention, raises interesting philosophical and ethical question with implications for drug development and regulation. One might also hypothesize that the action of microdosing and psychedelics relies on prior and continuously updating *belief* combining (perhaps synergistically) with a direct drug effect (Carhart-Harris et al., 2015; Carhart-Harris and Friston, 2019). Such a positive interaction could, in theory, be tested (Carhart-Harris et al., 2018), and if endorsed, this could be interpreted as implying that *belief* is an active component of the psychedelic treatment model, rather than a problematic confound.

In summary, here we created a novel, cost-effective, self-blinding, citizen-science methodology that enabled us to conduct the largest placebo-controlled study on psychedelics to-date and the first placebo-controlled examination of repeated psychedelic microdosing. Our findings confirm the anecdotal benefits of microdosing (improvements in a broad range of psychological measures); however, the results also suggest that the improvements are not due to the pharmacological action of microdosing, but are rather explained by the placebo effect (lack of significant between-groups effect).

## Figures and Tables

**Appendix 1—figure 1.**
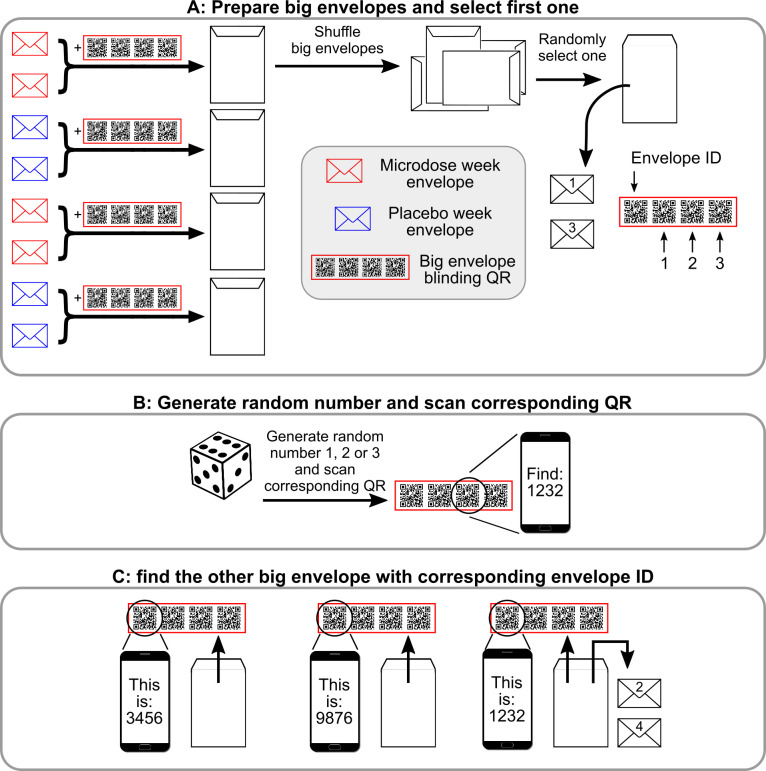
Semi-random drawing process during the self-blinding setup, which corresponds to panel B of Figure 2 in the main text. After the eight envelopes are prepared, pairs of microdose and placebo envelopes are placed inside big envelopes together with the corresponding ‘big envelope QR’ (a set of four QRS in the red frame) and then sealed. Once each big envelope is assembled, they are shuffled, and one is randomly selected. ‘1’ and ‘3’ are written on the two small envelopes inside (that contain the capsules and the weekly QR code, see Figure 2 of the main text) to designate that these will be used for weeks 1 and 3 (Panel **A**). Next, the other big envelope needs to be selected with the help of the big envelope QRs. Each big envelope QR has four QR codes: *envelope id* and *1,2,3*. First, a random number is generated between 1 and 3 (dice roll) and the corresponding QR is scanned (Panel **B**). Scanning displays a message ‘You need to find big envelope with ID XXXX’. Then, a random big envelope is opened and the ‘Envelope ID’ field is scanned from the big envelope QR inside. Scanning displays ‘This big envelope’s ID is YYYY’. This process is continued until big envelope XXXX is found. ‘2’ and ‘4’ are written on the two small envelopes inside to designate that these will be used for weeks 2 and 4.

**Appendix 1—figure 2.**
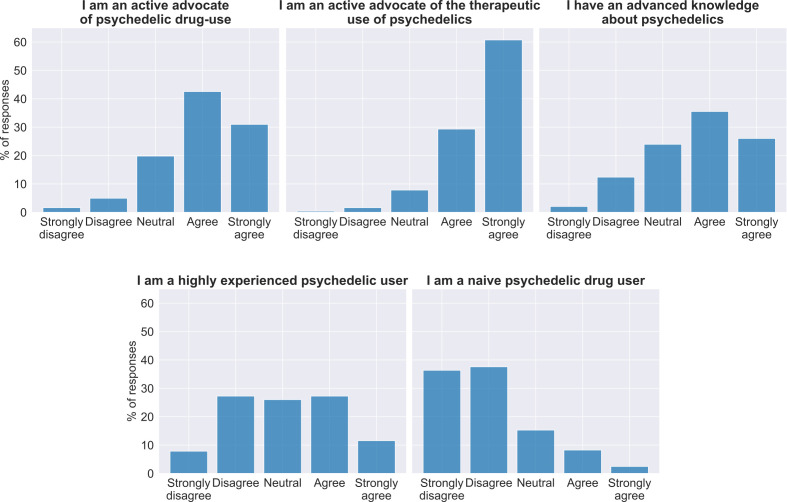
Attitude toward psychedelics in the sample at baseline. At the top of each panel is the item answered and below the bar graphs show the percentage of participant responses (n = 240).

**Appendix 1—figure 3.**
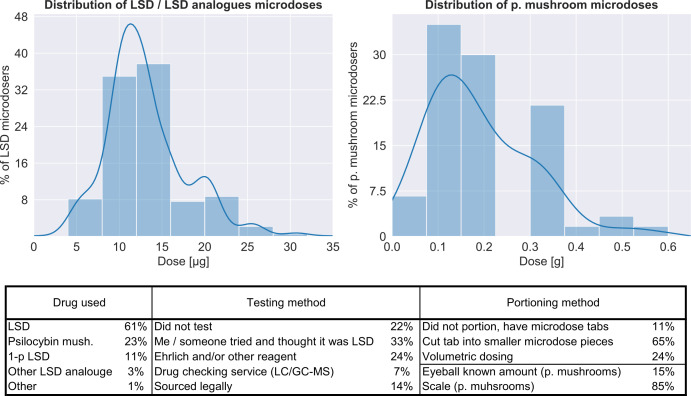
Characteristics of the microdoses used by participants. Top panel shows the distribution of doses for both LSD/LSD analogues (n = 180, 12.8 ± 5.5 μg) and psilocybin containing mushrooms (n = 56, 0.19 ± 0.12 g), bottom table provides additional information on the microdose preparation.

## Data Availability

All data is shared in CSV format at https://github.com/balazs1987/mcrds_public/tree/master/data (copy archived at https://archive.softwareheritage.org/swh:1:rev:43bb44cc8b6b79536e4b2afd0a7c724e90f137f7/) together with appropriate documentation.

## Additional files

Supplementary file 1. Sample demographics.

Supplementary file 2. Recreational drug use of sample.

Supplementary file 3. Baseline comparison of groups.

Supplementary file 4. Unadjusted accumulative scores.

Supplementary file 5. Adjusted accumulative differences.

Supplementary file 6. Accumulative model parameters.

Supplementary file 7. Acute model parameters.

Supplementary file 8. Adjusted strata differences.

Transparent reporting form

## Appendix 1

Description of measures (in alphabetical order) Big five personality inventory (B5) 64-item scale that captures each domain of the 5-factor model of personality (*Extraversion, Agreeableness, Conscientiousness, Neuroticism, and Opennes*), see [Costa and McCrae, 1992] for details. Our implementation included an additional *Intellect* dimension (DeYoung, 2015). Participants rated their agreement with items (e.g. ‘I make friends easily’) on a 5-point Likert scale and the score in the given dimension was the sum of the relevant item ratings. Personality dimensions are not additive; thus, each subscale was analyzed independently. Cognitive and affective mindfulness scale (CAMS, revised version) The revised CAMS is a 12-item scale measure of mindfulness. Items were rated on a 4-point scale (*Rarely/Not at all, Sometimes, Often,* and *Almost always*), see Feldman et al., 2007 for details. Total mindfulness score was used during analysis, which is a sum of the item scores. Cognitive performance score (CPS) Cognitive performance was measured both as an accumulative and as an acute outcome, because it was not clear from the microdosing anecdotes whether the reported cognitive benefits are present while under influence or after a period of microdosing. The Cambridge Bran Sciences (https://www.cambridgebrainsciences.com/) platform was used to collect cognitive performance data. To quantify cognitive performance, participants were tested in six tasks: *spatial span, paired associates, rotations, odd one out, spatial planning, and feature match* (see Hampshire et al., 2012 for details). Task scores were combined into a single CPS to quantify overall cognitive performance. To calculate the CPS, first the raw scores of each task were converted to a z-score. Then, to remove learning effects, the average score of the placebo group at the corresponding timepoint was subtracted:

$$Z_{ind,\;tp,\;t}^{adj}=Z_{ind,tp,t}-\;\left(\sum_{\begin{array}{c} inds\;in\;\\ PL\;group \end{array}}\frac{Z_{ind,\;tp,\;t}}{n_{PL,\;tp}}\right),$$

where $Z_{ind,tp,t}$ is the z-score of individual *ind* at timepoint *tp* on task *t* and $n_{PL,tp}$ is the number of individuals in the placebo group at timepoint *tp.* Finally, the CPS is calculated as the average adjusted z-score across the six tasks:

$${CPS}_{ind,tp}=\sum_{t}\frac{Z_{ind,\;\;tp,\;\;t}^{adj}}{6}\;.$$

In summary, CPS score is the z-score difference from the average of the placebo group who had the same number of previous opportunities to perform the tasks. Whenever the scores of the individual tasks are presented, the learning effects are always removed from the scores as described above (all steps prior to taking the average across the six subtasks). Daily effects of microdosing VASs (DEMS) DEMS is a set of self-constructed visual analogue scales designed to measure the acute effects of microdosing. Responses were collected on a scale of 0–100. The survey consisted of the following items with the corresponding [low; mid; high] anchor points:

- Please rate the intensity of the drug experience [No drug effects (placebo); Neutral/Average; Very intense drug effects]
- Please rate your mood for the day [Very negative; Neutral/Average; Very positive]
- Please rate your temper for the day (temper) [Very calm; Neutral/Average; Very tense]
- Please rate the (physical) energy you had today [Very low energy; Neutral/Average; Very high energy]
- Please rate your ability to keep focused today [Very easily distracted; Neutral/Average; Very focused]
- Please rate your creativity for today [Very uncreative; Neutral/Average; Very creative]

For all VAS items, the slider’s default position was the midpoint, but for a valid response the slider had to be moved. Demographics A self-constructed, general purpose 12-item questionnaire about sample demographics and mental health status; for details see Haijen et al., 2018. Green paranoid thought scales (GPTS) The 16-item ‘*social reference’* subscale was used, which focuses on social reference relevant to paranoia. Each item was rated on a 5-point Likert scale, and the sum score was used in analysis, see Green et al., 2008 for details. Note that higher scores indicate higher levels of paranoia, and thus, lower scores indicate improvements on this scale. Positive and negative affection scale (PANAS) A 20-item scale that consists of a number of words that describe different feelings and emotions. Each item is rated on a 5-point scale (*Not at All, A Little, Moderately, Quite a Bit,* and *Extremely*), see Watson et al., 1988 for details. PANAS has two subscales (*positive *and *negative*), and the final score used during analysis is the sum of the positive subscale minus the sum of the negative scale. Previous drug experiences and expectations (PDEE) A self-constructed 26-item questionnaire designed to measure the current and past recreational drug use intensity and participants’ relationship to psychedelics drugs, see *Attitude toward psychedelics and microdosing* section and (Haijen et al., 2018) for details. Quick inventory of depressive symptomatology (QIDS) The 16-item self-report version of the scale was used. It is a unidimensional scale, where each item has four response options that are assigned a numeric value, see Rush et al., 2003 for details. Sum of the item values was used in analysis, and lower score indicates fewer depressive symptoms. Ryff’s psychological well-being (RPWB) A 42-item instrument that consists of six subscales (*positive relations, personal growth, autonomy, environmental mastery, purpose in life*, and *self-acceptance*). To quantify well-being as a single outcome, the sum of the six subscales was used during analysis. The original scale uses a six-step rating (from *Strongly disagree* to *Strongly agree)*, see Ryff and Keyes, 1995 for details. In our online implementation a seven-step rating was used by accident (*Neutral* was added as an extra response option). To make our scores comparable with other studies, all RPWB scores have been rescaled by multiplying them with 6/7 and rounding it to the closest digit. Satisfaction with life (SWL) A 5-item unidimensional scale designed to measure judgment of one’s own life satisfaction, see Diener et al., 1985 for details. The scale uses a 7-point rating that ranges from *Strongly agree* to *Strongly disagree*; final score is the sum of item scores. Short suggestibility scale (SSS) SSS is a 21-item, unidimensional scale that quantifies an individual tendency to accept messages. Each item is rated on a 5-point scale (*Not at all, A little, Somewhat, Quite a bit, *and *A lot*), see Kotov et al., 2004 for details. The sum of the items was used in analysis. Social connectedness scale (SCS) SCS is a 8-item unidimensional scale that captures social belongingness, see Lee and Robbins, 1995 for details. Each item was rated on a 5-point Likert scale; final score is the sum of item scores. Spielberger’s state-trait anxiety inventory (STAIT) A 20-item scale where each item corresponds to a feeling or mental state (e.g. *‘I have disturbing thoughts’*), participants rate how often they felt that way on a 4-point scale (*Almost never, Sometimes, Often, *and *Almost always*), see Spielberger, 1983 for details. The appropriate sum of item scores (some items reverse scored) was used in analysis. Warwick–Edinburgh mental well-being (WEMWB) A 14-item unidimensional scale that covers both the feeling and functional aspects of mental well-being. Each item is rated on a 5-point scale (*None of the time, Rarely, Some of the time, Often,* and *All of the time*), see Tennant et al., 2007 for details. Sum of item scores was used in analysis. Additional information on the self-blinding setup and data collection For the MD group, all 4 weeks are microdose weeks, but the four variations of MD weeks (see Figure 1 for the variations) could be in any order. The order is determined by the random shuffling during setup. Similarly, for the HH group, the MD weeks during dose regime could be any two of the MD weeks (see Figure 1 for the four variations of the MD week). Furthermore, the sequence of MD and PL weeks has two variations, either MD-PL-MD-PL or PL-MD-PL-MD. During analysis no distinction was made between these variants. Variants of the setup were provided if psilocybin containing mushrooms or liquid was used. In the case of mushrooms (grinded powder), placebo capsules had to be filled with equal weight of non-psychoactive mushrooms, chaga (*Inonotus obliquus*) was recommended, otherwise the capsules could be distinguished based on their weight. For liquids, plastic vials had to be used to hold the substance and the placebo vials had to be filled with the same volume of liquid without the psychedelic component. Participants received an automated report after the completion of the long-term follow-up timepoint if all timepoints were completed. The report indicated what they have taken when, together with their CPSs (but not psychometric scores). As the report was sent after the completion of the last timepoint, its content did not affect any outcome measures. Participants were offered the option to construct their own dosing schedule with minimal restrictions, where the restrictions ensured the appropriate conditions for the acute and post-acute timepoints. Twelve participants choose this option. Seven of them maintained two microdoses/week (on MD weeks) and only moved the days of microdoses. Data from these individuals was included in the final analysis. The remaining five participants either had three (3) or a single (2) microdose during microdose weeks. To simplify the analysis, data from these five individuals was excluded. The time of completion was checked for every response and the data was discarded if the test was not completed in the appropriate time window. For the accumulative outcomes, tests had to be completed anytime during the week, while acute and post-acute tests had to be completed on the corresponding day. If a participant did not complete either the post-regime or the 4 weeks follow-up timepoints, an automated reminder was sent. If the test was completed within 48 hr, then the data was still considered valid. Participants were instructed not to complete tests if the dose schedule was not followed. Participants were allowed to withdraw from the study at any point. Their data was deleted if they explicitly asked for it, otherwise, the data was still included in the analyses. After scanning the QR codes, three participants had an invalid dose schedule (3PL weeks and a single MD week), likely due to errors during the setup. These three participants knew each other in real life and prepared the setup together, committing all the same mistake. Data from these individuals were discarded from the between-group analysis, as their dose regime does not conform to any of the groups, but data for the acute and post-acute measures was used. Attitude toward psychedelics and microdosing Appendix 1—figure 2 below shows the attitude toward psychedelic drugs in the sample. In general, the sample had a positive attitude toward psychedelics and consisted of knowledgeable users. Participants’ expectations about microdosing were measured at baseline using three visual analogue scales (VAS; 0–100) items.

1. How confident are you that the upcoming microdosing experience will have a long-lasting positive effect? (0 = not at all confident, 50 = somewhat confident, 100 = very confident),
2. At this point, how logical does the microdosing experience seem to you? (0 = not at all logical, 50 = somewhat logical, 100 = very logical),
3. At this point, how successfully do you think this experience will be in improving your overall well-being? (0 = not at all useful, 50 = somewhat useful, 100 = very useful).

These items were inspired by the credibility/expectancy questionnaire (Devilly and Borkovec, 2000), but were revised for the current study. An overall expectation score was calculated by calculating the mean of these scores. By this measure, the sample had highly positive expectations about microdosing, 68 ± 19. Microdose characteristics Blind breaking cues After the data collection was closed, a short survey was conducted among participants. It included one question about clues that lead to the guess, specifically: ‘When you guessed that your daily capsule was a microdose, what factors made you suspected that it was a microdose?’ (n = 166). The results were:

- Body/perceptual sensations only (24%)
- Mostly body/perceptual sensations, but some mental/psychological effects as well (31%)
- Equally because of body/perceptual sensations and mental/psychological effects (22%)
- Mostly mental/psychological effects, but some body/perceptual sensations as well (19%)
- Mental/psychological effects only (4%)

Below the question, there was a non-mandatory text box, where participants could have elaborated. Here, considerably fewer responses were received (n = 26). The consistent themes in the responses were muscle tingling/tension (n = 15), increased color saturation/visual warping (n = 12), and stomach stress/strain (n = 7). Only three text responses included effects related to the outcome measures (better mood/‘giggliness’). We note that due to a technical error, some participants were not invited to this survey, while a small fraction of participants were invited twice. Comparison to random guesser To compare participant’s performance to a random guesser, it was assumed that the random guesser only knows the ratio of PL/MD capsules (3/1) in the envelopes, and further assumes that this ratio remains constant through the experiment. Appendix 1—table 1 below shows the comparison of guessing performance of study participants per group and the random guesser.

**Appendix 1—table 1.** Comparison of guessing performance to a random guesser. X/Y (in the header columns) denotes condition X and guess Y. *Sensitivity* is the ratio of true positives to all positives (i.e. MD guessed and taken/MD taken), *specificity* is the ratio of true negatives to all negatives (i.e. PL guessed and taken/PL taken), while F1 is the harmonic mean of these metrics, used as a single number summary of accuracy. *Sensitivity* is not defined for the PL group, as they never take a microdose.

	True positive rate (MD/MD)	False negative rate (MD/PL)	False positive rate (PL/MD)	True negative rate (PL/PL)	Sensitivity	Specificity	F1-score
PL group participants	0	0	0.21	0.79	-	0.79	0
HH group participants	0.14	0.12	0.12	0.62	0.54	0.84	0.54
MD group participants	0.21	0.3	0.08	0.41	0.41	0.84	0.53
All participants	0.12	0.15	0.13	0.59	0.45	0.82	0.46
Random guesser	0.06	0.19	0.19	0.56	0.25	0.75	0.25

To calculate the threshold dose, above which participants guess significantly better than the random guesser defined above, numeric simulation of the setup and random guessing were conducted 200 times. Then, this distribution of random guess accuracies was tested with against the participant’s guess accuracy (with independent t-tests) who took equal or less than X µg. The dose was increased until the test was significant. With this method, the threshold dose was found to be 12 µg (*t*(317) = 1.92, p=0.05); above this dose participants tend to guess better than random. For this calculation, psilocybin containing mushroom doses were converted to LSD equivalents as described in the *Additional information on statistical models* section.
