# Supplementary material for: Self-blinding citizen science to explore psychedelic microdosing
Source: eLife. 2021 Mar 2;10:e62878. doi: 10.7554/eLife.62878 (PMC7925122; doi:10.7554/eLife.62878)
Supplement: Transparent reporting form [file elife-62878-transrepform.docx]

***eLife’s* transparent reporting form**

We encourage authors to provide detailed information *within their submission* to facilitate the interpretation and replication of experiments. Authors can upload supporting documentation to indicate the use of appropriate reporting guidelines for health-related research (see [EQUATOR Network](http://www.equator-network.org/%20)), life science research (see the [BioSharing Information Resource](https://biosharing.org/)), or the [ARRIVE guidelines](http://www.plosbiology.org/article/info:doi/10.1371/journal.pbio.1000412) for reporting work involving animal research. Where applicable, authors should refer to any relevant reporting standards documents in this form.

If you have any questions, please consult our Journal Policies and/or contact us: [editorial@elifesciences.org](mailto:editorial@elifesciences.org).

**Sample-size estimation**

- You should state whether an appropriate sample size was computed when the study was being designed
- You should state the statistical method of sample size computation and any required assumptions
- If no explicit power analysis was used, you should describe how you decided what sample (replicate) size (number) to use

Please outline where this information can be found within the submission (e.g., sections or figure legends), or explain why this information doesn’t apply to your submission:

There was no same size calculation prior to conducting the study due to the online nature of the study. Participants needed to get involved on their own initiative, so the sample size was not decided by the research team, but rather dictated by the number of people who signed up and completed the study. We made significant effort to make the sample size as high as possible by engaging the psychedelics community through the available channels. For details related to the online nature of the study and the recruitment strategy, see “Recruitment and inclusion criteria” section of the Methods.

**Replicates**

- You should report how often each experiment was performed
- You should include a definition of biological versus technical replication
- The data obtained should be provided and sufficient information should be provided to indicate the number of independent biological and/or technical replicates
- If you encountered any outliers, you should describe how these were handled
- Criteria for exclusion/inclusion of data should be clearly stated
- High-throughput sequence data should be uploaded before submission, with a private link for reviewers provided (these are available from both GEO and ArrayExpress)

Please outline where this information can be found within the submission (e.g., sections or figure legends), or explain why this information doesn’t apply to your submission:

Due to the nature of the study there was no replication. All details related to data exclusion/inclusion is described in the “Additional information on the self-blinding setup and data collection” of the supplementary materials.

**Statistical reporting**

- Statistical analysis methods should be described and justified
- Raw data should be presented in figures whenever informative to do so (typically when N per group is less than 10)
- For each experiment, you should identify the statistical tests used, exact values of N, definitions of center, methods of multiple test correction, and dispersion and precision measures (e.g., mean, median, SD, SEM, confidence intervals; and, for the major substantive results, a measure of effect size (e.g., Pearson's r, Cohen's d)
- Report exact p-values wherever possible alongside the summary statistics and 95% confidence intervals. These should be reported for all key questions and not only when the p-value is less than 0.05.

Please outline where this information can be found within the submission (e.g., sections or figure legends), or explain why this information doesn’t apply to your submission:

Statistical methods and their justification are described in the “Statistical analysis” section of Methods. Some additional details are provided in the “Additional information on statistical models” of the supplementary materials. All sample size, mean and effect size measures are described in the results section and the appropriate figure captions. This information for all subscale measures (mean and effect size) can be found in the supplementary information (S5, S6 and S9) as some of these are not discussed in the main manuscript.

(For large datasets, or papers with a very large number of statistical tests, you may upload a single table file with tests, Ns, etc., with reference to sections in the manuscript.)

**Group allocation**

- Indicate how samples were allocated into experimental groups (in the case of clinical studies, please specify allocation to treatment method); if randomization was used, please also state if restricted randomization was applied
- Indicate if masking was used during group allocation, data collection and/or data analysis

Please outline where this information can be found within the submission (e.g., sections or figure legends), or explain why this information doesn’t apply to your submission:

Group allocation was determined by a random number generated during the self-blinding setup procedure. Participants were instructed to use either a dice or the random.org website to generate the required random number. Knowledge of this number did not allow to gain knowledge of group allocation, it simply determined which of the QR codes participants need to use during self-blinding. Details of self-blinding, which includes the group allocation, can be found in Figure 1 and S1.

**Additional data files (“source data”)**

- We encourage you to upload relevant additional data files, such as numerical data that are represented as a graph in a figure, or as a summary table
- Where provided, these should be in the most useful format, and they can be uploaded as “Source data” files linked to a main figure or table
- Include model definition files including the full list of parameters used
- Include code used for data analysis (e.g., R, MatLab)
- Avoid stating that data files are “available upon request”

Please indicate the figures or tables for which source data files have been provided:

Table S7 and S8 in the supplementary materials list all the parameters of all the statistical models that were used during analysis. All data is shared in CSV format at <https://github.com/balazs1987/mcrds_public/tree/master/data> together with appropriate documentation. The repository also contains python, R and SAS scripts that were used to produce statistical findings from the manuscript. See documentation within the repository for details.
