# Supplementary material for: Self-blinding citizen science to explore psychedelic microdosing
Source: eLife. 2021 Mar 2;10:e62878. doi: 10.7554/eLife.62878 (PMC7925122; doi:10.7554/eLife.62878)
Supplement: Peer review history

# Decision letter

**DOI:** 10.7554/eLife.62878.sa1

**Affiliations:** University of MarylandUnited States; United States; Netherlands

In the interests of transparency, eLife publishes the most substantive revision requests and the accompanying author responses.

**Acceptance summary:**

The field knows astonishingly little about psychedelic microdosing given the prevalence of the practice. This study assessed the acute and long term effects of microdosing on subjective and cognitive measures, with a self-blinding method allowing to examine potential placebo effects.

**Decision letter after peer review:**

Thank you for submitting your article "Self-blinding citizen science to explore in psychedelic microdosing" for consideration by *eLife*. Your article has been reviewed by three peer reviewers, and the evaluation has been overseen by Alexander Shackman as the Reviewing Editor and Chris Baker as the Senior Editor.

The reviewers have discussed the reviews with one another and the Reviewing Editor has drafted this decision to help you prepare a revised submission.

As the editors have judged that your manuscript is of interest, but as described below that additional experiments are required before it is published, we would like to draw your attention to changes in our revision policy that we have made in response to COVID-19 (https://elifesciences.org/articles/57162). First, because many researchers have temporarily lost access to the labs, we will give authors as much time as they need to submit revised manuscripts. We are also offering, if you choose, to post the manuscript to bioRxiv (if it is not already there) along with this decision letter and a formal designation that the manuscript is "in revision at *eLife*". Please let us know if you would like to pursue this option. (If your work is more suitable for medRxiv, you will need to post the preprint yourself, as the mechanisms for us to do so are still in development.)

Summary:

This study assessed the acute and long term effects of microdosing on subjective and cognitive measures, with a self-blinding method allowing to examine potential placebo effects.

The reviewers and I were generally quite enthusiastic about the study and report:

• The manuscript describes a complex, ingeniously designed online study undertaken

to examine of the acute, post-acute, and enduring effects of psychedelic microdosing;

• At first, I had my doubts when I read the words “self-blinding”, but became very enthusiastic about this method as it is very well thought-out and a very good addition to the literature;

• Clever use of the Citizen Science Platform;

• Authors clearly took great care in planning and conducting the trial;

• Transparent reporting;

• Paper seems well polished;

• Writing is clear and methodical.

Nevertheless, several concerns somewhat dampened our collective enthusiasm:

• Blinding.

o More clarity is needed in the Materials and methods about how participants were blinded and guided to know what a microdose was. This is important to include in the main text. I appreciate the blinding schematics provided by the authors. In addition, a brief description of Citizen Science would be useful in the main text.

o Please clarify that participants who broke blind did not have their data included, or please clarify why. Also please report the frequency of how many participants broke blind.

• Data availability.

In theory, data is publicly available on their website, but we encountered difficulties accessing it. Please correct this error or address whether it become available upon acceptance of the manuscript.

# Reply: Author response

**DOI:** 10.7554/eLife.62878.sa2

[…] Several concerns somewhat dampened our collective enthusiasm:

• Blinding.

o More clarity is needed in the Materials and methods about how participants were blinded and guided to know what a microdose was. This is important to include in the main text. I appreciate the blinding schematics provided by the authors. In addition, a brief description of Citizen Science would be useful in the main text.

We have added a new subsection to the Materials and methods, titled “Self-blinding setup procedure”, which provides a high-level overview of the setup process. This section provides a summary of the procedure for the readers, while further details can be found in the schematic description. The text of this subsection reads as follows:

“A high-level overview of self-blinding is provided here, for a detailed illustration see Figure 2. […] With the QR codes in place, it is possible for the experimenter to recover knowledge of capsule types after randomization without revealing that information to participants.”

Furthermore, we have also added a “Microdose preparation” subsection to the Materials and methods, detailing the directions given to participants. The new section reads:

“Participants were allowed to use any psychedelic substance to microdose with. The microdose dose, i.e. the amount of substance to use as a microdose, was not defined for participants, rather they were instructed to use a microdose dose that they would use outside the study. […] Lastly, this study was not a clinical trial and therefore from a regulatory perspective not allowing for control over and/or directing about drug doses.”

o Please clarify that participants who broke blind did not have their data included, or please clarify why. Also please report the frequency of how many participants broke blind.

Data when participants breaking blind are included in all analysis, in agreement with conventional psychiatric trials that also include such data. We note that blinding is rarely assessed in clinical studies [Baethge, 2013], so examples are limited, nonetheless see [Chen, 2011] or [Rabkin, 1986] for antidepressant studies with guess data. In neither case break blind datapoints are removed from the main analysis, similarly to our study.

As for the break blind rate, we first note that we asked participants to guess their treatment daily (for every day of the dose period). Hence, we can only talk about the % of days when participants break blind (rather than the % of participants) – the same participant could break blind on some days, while not on others. We have updated the subsections “Blind breaking and collection of guess data” (Materials and methods) and “Blinding integrity” (Results) to avoid possible misunderstandings. In particular, now the “Blind breaking and collection of guess data” subsection contains the following sentence:

“In the discussion of our results, the term “break blind” indicates that the participant guessed the capsule correctly for the day (acute outcomes) or week (post-acute outcomes).”

Participants broke blind on 72% of their daily assessments, as it is already reported with details in the “Blinding integrity” subsection in the Results.

• Data availability.

In theory, data is publicly available on their website, but we encountered difficulties accessing it. Please correct this error or address whether it become available upon acceptance of the manuscript.

We checked and the data together with the documentation is publicly available in the data/ folder of the repository we shared. To avoid any misunderstandings, we updated the Transparent reporting document and now a new link is provided, which directly points to the data folder (rather than the repository itself). Furthermore, we removed code that is not relevant to reproducing the results, hoping to make it easier to navigate the codebase. Please let us know if the issue persists.
